# Intrahepatic distribution of nerve fibers and alterations due to fibrosis in diseased liver

**DOI:** 10.1371/journal.pone.0249556

**Published:** 2021-04-14

**Authors:** Kei Mizuno, Hiroaki Haga, Kazuo Okumoto, Kyoko Hoshikawa, Tomohiro Katsumi, Taketo Nishina, Takafumi Saito, Hideki Katagiri, Yoshiyuki Ueno

**Affiliations:** 1 Department of Gastroenterology, Yamagata University Faculty of Medicine, Yamagata, Japan; 2 Japan Agency for Medical Research and Development, Core Research for Evolutional Science and Technology (CREST), Tokyo, Japan; 3 Department of Metabolism and Diabetes, Tohoku University Graduate School of Medicine, Sendai, Japan; Nihon University School of Medicine, JAPAN

## Abstract

Autonomic nerve fibers in the liver are distributed along the portal tract, being involved in the regulation of blood flow, bile secretion and hepatic metabolism, thus contributing to systemic homeostasis. The present study investigated changes in hepatic nerve fibers in liver biopsy specimens from patients with normal liver, viral hepatitis and non-alcoholic steatohepatitis, in relation to clinical background. The areal ratio of nerve fibers to the total portal area was automatically calculated for each sample. The nerve fiber areal ratios (NFAR) for total nerve fibers and sympathetic nerve fibers were significantly lower in liver affected by chronic hepatitis, particularly viral hepatitis, and this was also the case for advanced liver fibrosis. However, the degree of inflammatory activity did not affect NFAR for either whole nerves or sympathetic nerves. Comparison of samples obtained before and after antiviral treatment for HCV demonstrated recovery of NFAR along with improvement of liver fibrosis.

## Introduction

The relationship between the gastrointestinal tract and the autonomic nervous system has been intensively investigated, especially in the context of digestive tract motility, fluid transport, and the endocrine system [1]. The liver is innervated by both sympathetic and parasympathetic nerves. The sympathetic fibers are postganglionic fibers connected to the celiac ganglia and superior mesenteric ganglia, derived from preganglionic fibers exiting the spinal cord at T7-T12, whereas the parasympathetic fibers branch from the vagus nerve and are thought to innervate either directly as preganglionic fibers derived from the dorsal motor nucleus of the brainstem or via synapses in the ganglia of the hepatic hilus [2]. However, the location of the hepatic ganglia remains unknown. These nerve fibers enter the liver from the hepatic hilus, and are distributed along the portal triad. In the human liver, sympathetic fibers extend to the liver lobules, whereas the parasympathetic nerves are distributed only in the portal tract [3]. Although certain variations exist among animal species, intrahepatic nerve fibers are reported to surround the hepatic artery, portal vein, and bile duct in most cases [4].

Among hepatic nerve fibers, afferent fibers are responsible for reporting information such as osmotic pressure and the hepatic concentrations of glucose and lipids to the brain [2, 5–7], whereas efferent fibers transmit orders from the brain to regulate blood flow, bile secretion, lipid synthesis, and glycogenesis for maintenance of body homeostasis [2, 8–10]. Although both blood vessels and bile duct are anatomically reconstructed in liver transplantation, nerves remain disconnected. Liver transplant recipients are reported to frequently develop a variety of systemic conditions such as obesity, lipid abnormalities, hypertension, and diabetes mellitus [11, 12]. We have hypothesized that this systemic dysregulation could be related to hepatic denervation.

Treatments for viral hepatitis recently have been advanced, such as direct-acting antivirals (DAAs) for hepatitis C virus (HCV) and novel nucleos(t)ide analogs for hepatitis B virus (HBV). Eradication of the hepatitis virus is well-known to result in improvement of fibrosis and reduce the likelihood of carcinogenesis [13–15]. Conversely, nonalcoholic steatohepatitis (NASH), a hepatic phenotype of metabolic syndrome, has been a focus of interest in recent decades. To our knowledge, no approved pharmacologic therapies exist for NASH. In mice given a methionine-choline-deficient diet (an animal model of NASH), vagotomy induces a more profound histological derangement relative to nonvagotomized controls [16]. However, no previous study has examined changes in intrahepatic nerve fibers during NASH development, and even in viral hepatitis, evaluation of changes in intrahepatic nerve fibers remains insufficient.

Intrahepatic nerve fibers are reported to be involved in hepatic fibrosis and liver regeneration. Hepatic stellate cells (HSCs) are activated during liver injury, and are transformed into myofibroblast-like cells that produce collagen fibers [17]. HSCs possess adrenoceptors and the growth of HSCs is accelerated by sympathetic stimulation, a feature involved in the progression of hepatic fibrosis [18]. Although hepatic progenitor cells are activated and differentiate into hepatocytes after severe liver injury, their proliferation can be enhanced by inhibition of the sympathetic nervous system, resulting in amelioration of hepatic injury [18, 19]. In contrast, vagotomy impairs liver regeneration [20, 21].

Thus, elucidating the relationship between liver disease and intrahepatic nerve fibers that regulate various liver functions may contribute to the development of treatment for liver disease.

To elucidate changes in the distribution of nerve fibers in a variety of chronic liver diseases, we investigated the distribution of nerve fibers in liver biopsy specimens in terms of both whole nerve fibers and sympathetic fibers, and also in relation to clinical factors. Furthermore, using liver biopsy specimens before and after antiviral treatment for HCV, we investigated chronological changes in nerve fibers during improvement of hepatic fibrosis.

## Methods

### Subjects and materials

The liver specimens examined in this study were from 85 patients who had undergone liver biopsy at Yamagata University Hospital between 2006 and 2017. The diagnoses included normal liver (n = 5), viral hepatitis (n = 45: HBV/HCV = 20/35), and NASH (n = 35). Patients diagnosed with moderate-to-severe fatty liver disease by abdominal ultrasonography were excluded from the viral hepatitis cases. Three cases of chronic hepatitis C were compared before and after treatment: interferon monotherapy in two cases and peginterferon and ribavirin combination therapy in one.

Before liver biopsy, all patients had provided written informed consent for sample preservation and use for research. An outline of the study was published on our website, and patients confirmed their intention to participate or withdraw from the study by responding to an opt-out request for exclusion. The study was conducted in accordance with relevant guidelines [22, 23] and regulations under protocols approved by the Ethics Committee of Yamagata University Faculty of Medicine (IRB Accession number: 2018–74).

### Histochemical and immunohistochemical staining

Formalin-fixed paraffin-embedded liver biopsy specimens were cut into sections 3 μm thick, deparaffinized, and subjected to antigen retrieval at 98°C for 20 minutes. After blocking with 3% hydrogen peroxide for 5 min, the sections were incubated for 50 min at room temperature with primary antibodies: anti-protein gene product (PGP) 9.5 rabbit polyclonal antibody (1:150, cat# ADI-905-520-1, Enzo Life Science, NY, USA) for whole nerve fibers and anti-tyrosine hydroxylase (TH) rabbit polyclonal antibody (1:250, cat# ab112, Abcam, Cambridge, UK) for sympathetic nerve fibers. As the secondary antibody, Histofine Simple Stain MAX-PO(MULTI) (cat# 424151, Nichirei Bioscience, Tokyo, Japan) was used. All samples were subjected to final color development using 3, 3’-diaminobenzidine (DAB).

Fibrosis and inflammation were evaluated according to the METAVIR scoring system [24] and Brunt’s classification [25] using hematoxylin-eosin staining, Elastica-Masson staining, and silver staining.

### Histological and statistical analysis

**Fig 1:**
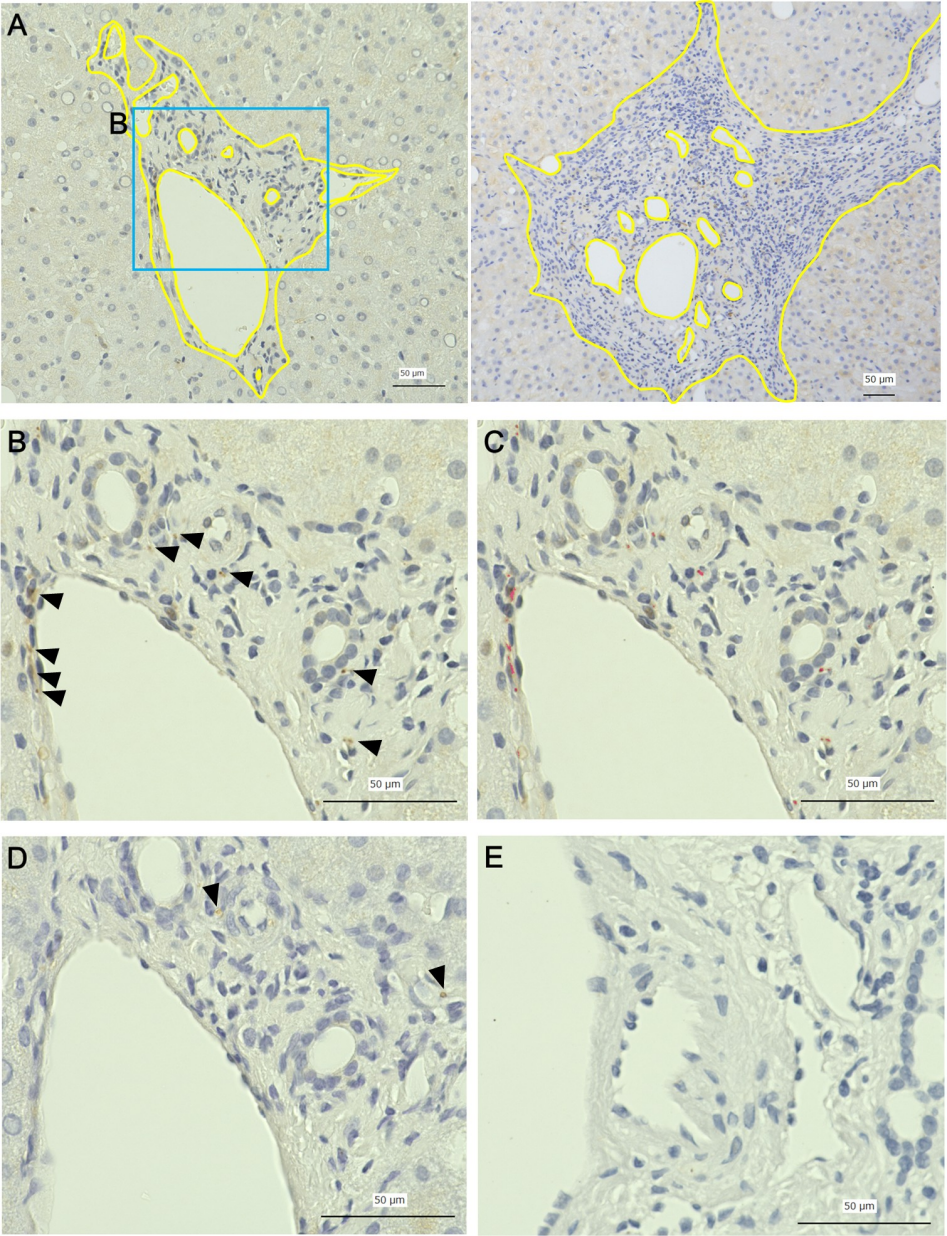
Quantitative measurement of intrahepatic nerve fibers. A: representative picture of the portal area (left: a case of mild fibrosis, right: a case of advanced fibrosis, yellow line: outer border of the portal area and border of the lumen), B: immunohistochemical staining for PGP9.5 (arrowheads), C: automatically detected area of immunohistochemically positive nerve fibers (red area), D: immunohistochemical staining for TH (arrowheads), E: negative control. Nerve fiber area ratio (NFAR) was obtained using the formula: NFAR = area of immunohistochemically positive nerve fibers (μm^2^)/ (portal area–vessel luminal area). Scale bars = 50 μm.

The immunohistochemically stained specimens were observed with a microscope (BZ-X710, Keyence, Osaka, Japan), and image analysis software (BZ-H3C/Hybrid Cell Count, Keyence, Osaka, Japan) was used to measure the nerve fiber axonal cross-sectional area and the portal area excluding all vessel lumina. The average areal ratio of nerve fibers to the total portal area excluding the vessel lumina was calculated for each sample to estimate the amount of nerve fibers (Fig 1). These nerve fiber areal ratios (NFAR) were compared among samples of normal liver, viral hepatitis and NASH, and also among the degree of inflammation, the degree of fibrosis, and the level of serum lipids and plasma glucose. The degree of inflammation was evaluated using the METAVIR scoring system and the level of serum ALT. The degree of fibrosis was assessed using the METAVIR scoring system, platelets, FIB-4 index calculated from age, serum AST, serum ALT, and platelets, and Mac-2 binding protein glycosylation isomer (M2BPGi) [26–28]. M2BPGi was measurable in only 75 cases as it had been additionally measured using serum collected on admission for liver biopsy and stored frozen at -70°C. Serum triglycerides, total cholesterol, and fasting plasma glucose were used as metabolism-related laboratory data, then divided into high-value and low-value groups and compared. Comparison between normal liver, viral hepatitis, and NASH, and comparison by pathological classification were performed by one-way ANOVA and Tukey’s multiple comparison test. Comparison by laboratory test values was performed by *t* test. Differences at P < 0.05 were considered significant. For statistical analysis, GraphPad Prism 7 (GraphPad Software, CA, USA) was used.

## Results

### Patient characteristics

The characteristics of the patients at the time of biopsy are shown in Table 1. The average ages were in their 50s and 60s, and there was no significant inter-group difference in the gender ratio. The samples of normal liver were from patients with muscle disease who had undergone liver biopsy for investigation of serum AST or ALT abnormalities, or represented the background liver tissue from patients who had undergone biopsy of liver tumors. Accordingly, such patients may have had poor nutrition, with low serum albumin levels. There were no differences in serum AST and ALT levels among the normal liver, NASH and chronic viral hepatitis groups. In the NASH group, the levels of fasting plasma glucose and HbA_1c_ were significantly higher, and the triglyceride level tended to be higher than in the other two groups. The platelet count was significantly lower in the patients with viral hepatitis, especially those with hepatitis C. The FIB-4 index and the level of M2BPGi, which are liver fibrosis markers [26–28], were low in the normal liver group with no pathological evidence of fibrosis, but high in the chronic hepatitis groups, particularly in patients with viral hepatitis.

**Table 1:** Patient characteristics.

	Normal (n = 5)	Viral Hepatitis (n = 45)			NASH (n = 35)	*P* value			
		Total (n = 45)	HBV (n = 20)	HCV (n = 25)			Normal vs Viral	Normal vs NASH	Viral vs NASH
Age (y.o.), mean ± SD	54.0 ± 18.3	57.3 ± 14.7	50.4 ± 17.7	62.8 ± 8.9	58.5 ± 14.0	0.799*	0.882^†^	0.798^†^	0.930^†^
Male, n (%)	2 (40.0)	24 (53.3)	13 (65.0)	11 (44.0)	13 (37.1)	0.349^‡^			
A0/1/2/3	5/0/0/0	0/22/14/9	0/11/7/2	0/11/7/7	0/16/13/6				
F0/1/2/3/4	5/0/0/0/0	0/19/9/10/7	0/9/4/5/2	0/10/5/5/5	0/14/6/9/6				
G1/2/3					19/11/5				
S1/2/3/4					16/4/9/6				
Alb (g/dL), mean ± SD	3.6 ± 0.7	4.0 ± 0.4	3.9 ± 0.5	4.0 ± 0.3	4.1 ± 0.5	0.041*	0.341^†^	0.070^†^	0.171^†^
AST (U/L), mean ± SD	27.2 ± 14.3	67.2 ± 64.1	79.3 ± 77.3	57.5 ± 50.8	70.3 ± 28.5	0.205*	0.219^†^	0.181^†^	0.960^†^
ALT (U/L), mean ± SD	36.4 ± 22.9	95.6 ± 119.9	124.9 ± 155.3	72.1 ± 77.2	86.2 ± 48.3	0.403*	0.375^†^	0.507^†^	0.896^†^
TG (mg/dL), mean ± SD	87.6 ± 35.1	109.5 ± 74.5	95.6 ± 39.9	119.6 ± 91.3	132.2 ± 60.4	0.202*	0.770^†^	0.353^†^	0.306^†^
TC (mg/dL), mean ± SD	200.6 ± 41.0	167.0 ± 34.3	175.2 ± 27.5	161.2 ± 37.8	184.2 ± 39.9	0.046*	0.142^†^	0.628^†^	0.111^†^
FPG (mg/dL), mean ± SD	85.8 ± 1.9	98.7 ± 19.8	97.3 ± 21.6	99.7 ± 18.7	123.0 ± 35.0	0.0002*	0.573^†^	0.014^†^	0.0005^†^
HbA1c (%), mean ± SD	5.6 ± 0.4	5.6 ± 0.7	5.8 ± 1.0	5.5 ± 0.5	6.5 ± 1.0	0.0002*	0.990^†^	0.065^†^	0.0002^†^
Plt ×10^3^ (/μL), mean ± SD	265.0 ± 40.9	153.8 ± 66.5	171.3 ± 75.2	139.7 ± 56.3	204.6 ± 89.4	0.0009*	0.007^†^	0.225^†^	0.011^†^
FIB-4 index, mean ± SD	1.1 ± 0.7	3.9 ± 5.0	3.9 ± 6.9	3.8 ± 2.8	3.1 ± 2.1	0.263*	0.286^†^	0.542^†^	0.619^†^
M2BPGi (C.O.I.), mean ± SD	0.4 ± 0.2 (n = 4)	3.4 ± 3.2 (n = 42)	2.8 ± 3.2 (n = 17)	3.9 ± 3.2 (n = 25)	1.8 ± 2.0 (n = 29)	0.014*	0.095^†^	0.619^†^	0.039^†^

Characteristics of patients with normal liver (n = 5), viral hepatitis (n = 45: HBV/HCV = 20/25), and NASH (n = 35). A: grading of inflammation evaluated using the METAVIR scoring system, F: staging of fibrosis assessed using the METAVIR scoring system, G: grading of inflammation evaluated using Brunt’s classification, S: staging of fibrosis assessed using Brunt’s classification, Alb: albumin, AST: aspartate aminotransferase, ALT: alanine aminotransferase, TG: triglyceride, TC: total cholesterol, FPG: fasting plasma glucose, Plt: platelet count, M2BPGi: Mac-2 binding protein glycosylation isomer (normal range: <1.00).

* one-way ANOVA

^†^ Tukey’s multiple comparison test

^‡^ χ^2^ test.

### Comparison among liver diseases

The liver specimens were evaluated pathologically in accordance with the METAVIR scoring system [24]. Normal liver was A0, F0 without inflammatory cell infiltration or fibrosis, and chronic hepatitis was classified as either A1 to A3 or F1 to F4. Both the viral hepatitis and NASH cases tended to be relatively mild (Table 1).

**Fig 2:**
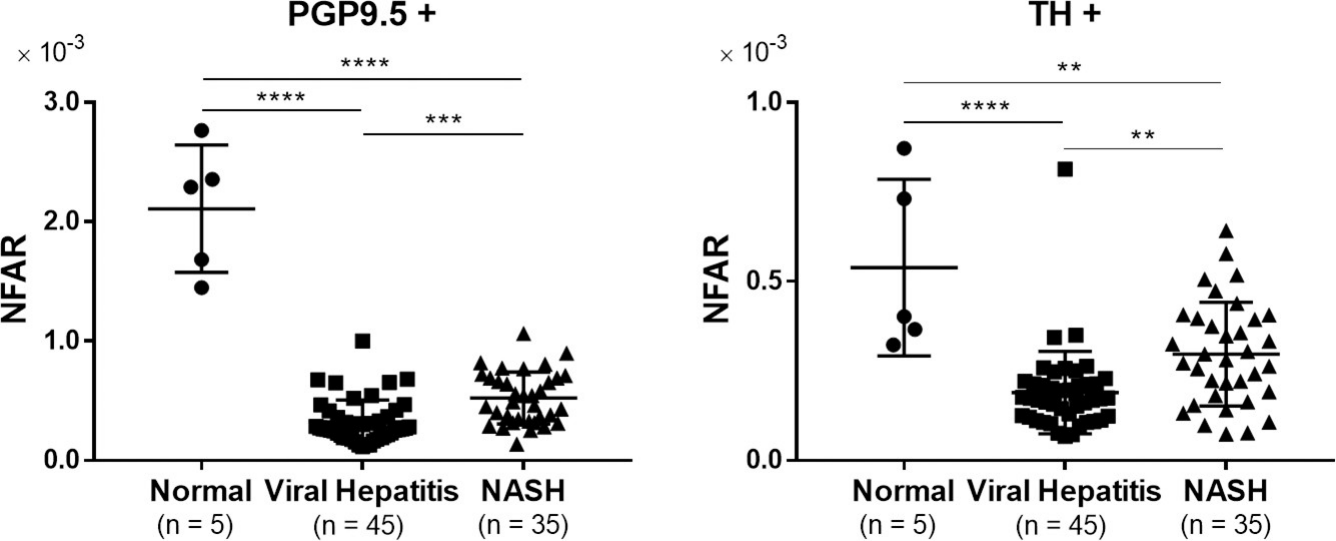
Comparison of NFAR among liver diseases. Comparison of NFAR for whole nerve fibers (PGP9.5+) and sympathetic nerve fibers (TH+). Data were analyzed by one-way ANOVA and Tukey’s multiple comparison test. ** P < 0.01, *** P < 0.001, **** P < 0.0001.

NFAR comparisons among the various types of liver disease are shown in Fig 2. The NFAR for normal liver was significantly higher than that for viral hepatitis and NASH in terms of both whole nerve fibers and sympathetic nerve fibers (PGP9.5+ viral: P < 0.0001, PGP9.5+ NASH: P < 0.0001, TH+ viral: P < 0.0001, TH+ NASH: P = 0.004), suggesting that chronic hepatitis–particularly viral hepatitis–leads to a reduction of intrahepatic nerve fibers.

### Relationship between fibrosis and NFAR

**Fig 3:**
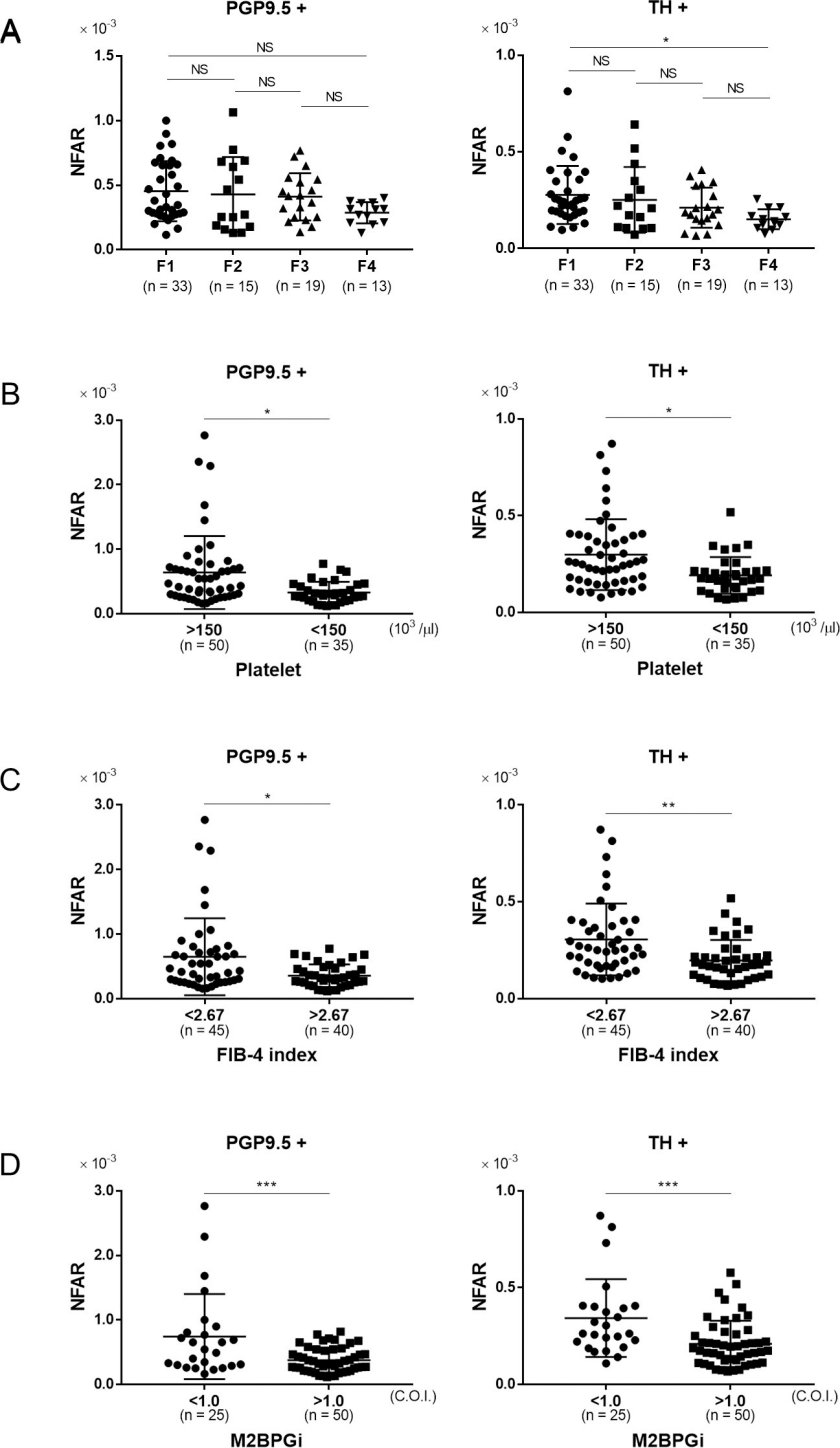
The relationship between fibrosis and NFAR. Comparison of NFAR for whole nerve fibers (PGP9.5+) and sympathetic nerve fibers (TH+). A: mild fibrosis (F1) to cirrhosis (F4), B: higher platelet count (≥ 150×10^3^/μl) vs. lower platelet count (< 150×10^3^/ μl), C: lower FIB-4 index (< 2.67) vs. higher FIB-4 index (≥ 2.67), D: lower Mac-2 binding protein glycosylation isomer (M2BPGi) level (< 1.5 C.O.I.) vs. higher M2BPGi level (≥ 1.5 C.O.I.). Data were analyzed by unpaired t test. * P < 0.05, ** P < 0.01, *** P < 0.001.

In order to clarify the relationship between liver fibrosis and intrahepatic NFAR, we performed a comparison between four groups: those diagnosed as F1 (mild fibrosis) to those as F4 (liver cirrhosis) (Fig 3A). In the F4 group, a significant decrease in NFAR compared with F1 was observed for sympathetic nerve fibers (F1 vs. F2: P = 0.92, F1 vs. F4: P = 0.02, F2 vs. F3: P = 0.81, F3 vs. F4: P = 0.60). Although there was no significant difference in whole nerve fibers, it tended to be lower in the F4 group (F1 vs. F2: P = 0.98, F1 vs. F4: P = 0.09, F2 vs. F3: P = 0.99, F3 vs. F4: P = 0.39). Comparison between patients with a platelet count of ≥ 150×10^3^/μl and < 150×10^3^/μl (the latter expected to have advanced fibrosis) showed that the latter had a significantly decreased NFAR in terms of both whole nerve fibers and sympathetic nerve fibers (Fig 3B, PGP9.5+: P = 0.02, TH+: P = 0.01). Although the criteria for the FIB-4 index differ depending on the etiology of liver disease, we divided the patients into two groups: < 2.67 and ≥ 2.67 [29]. The group with a higher FIB-4 index (considered to have advanced fibrosis) had a significantly reduced NFAR in comparison to the group with a lower FIB-4 index (Fig 3C, PGP 9.5+: P = 0.02, TH+: P < 0.008). Furthermore, although M2BPGi has a different cut-off index (COI) for estimation of hepatic fibrosis among various liver diseases, cases with a M2BPGi COI of ≥ 1.5 could be considered to have more advanced fibrosis than those with a COI of < 1.5 [30]. The NFAR was significantly decreased in the high M2BPGi group relative to the low group (Fig 3D, both P < 0.001). Overall, the data for both indices indicated that NFAR was significantly decreased in patients with advanced fibrosis.

### Relationship between inflammation and NFAR

**Fig 4:**
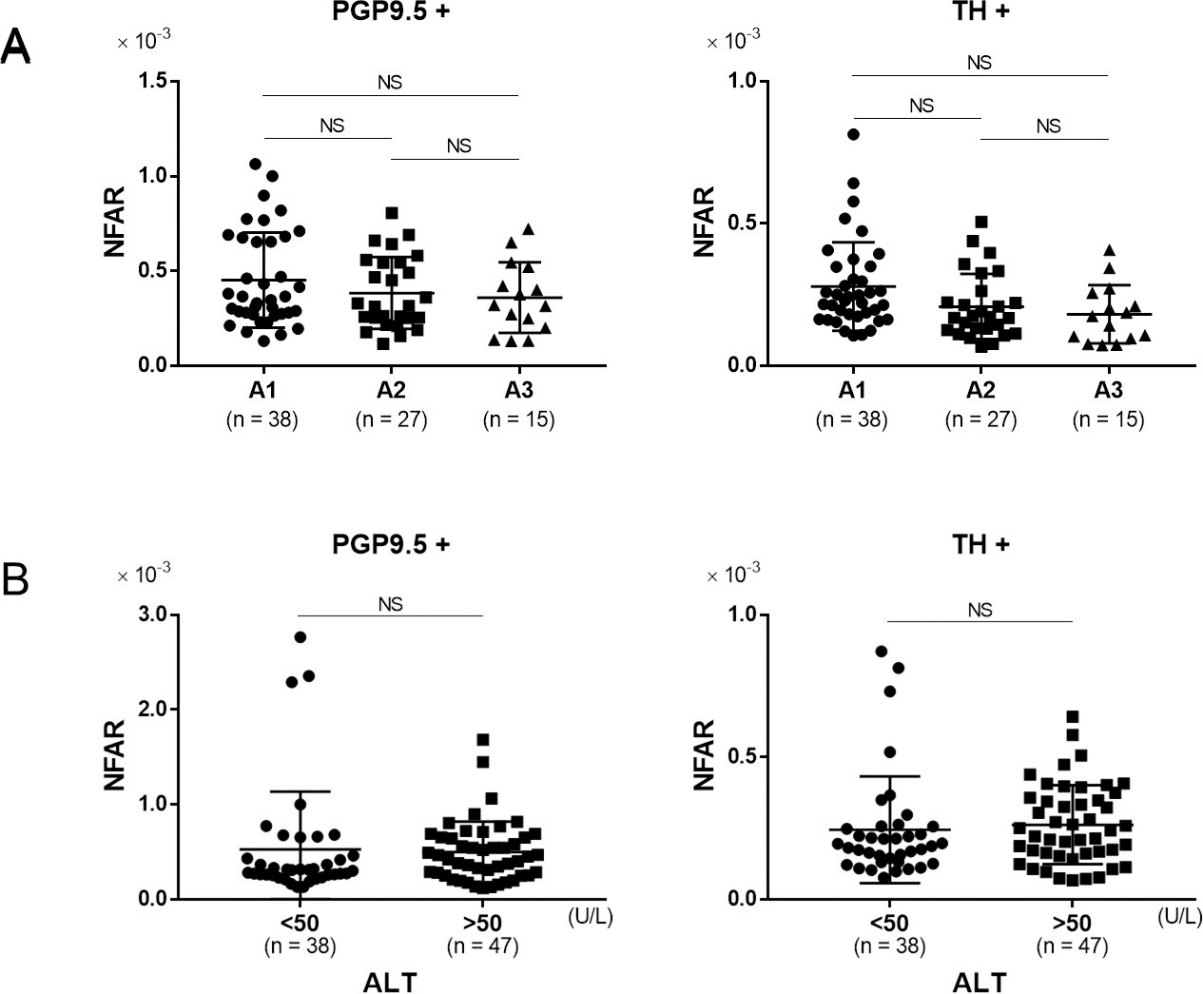
The relationship between inflammation and NFAR. Comparison of NFAR in for whole nerve fibers (PGP9.5+) and sympathetic nerve fibers (TH+). A: mild inflammation (A1) to severe inflammation (A3), B: lower alanine aminotransferase (ALT) level (< 50 U/L) vs. higher ALT level (≥ 50 U/L). Data were analyzed by unpaired t test. NS: not significant.

In order to evaluate the relationship between inflammation and NFAR, we performed comparison between patients diagnosed with A1 (mild inflammation) to those with A3 (severe inflammation). Although NFAR for both whole nerve fibers and sympathetic nerve fibers tended to be lower for A3, the difference was not statistically significant (Fig 4A, PGP9.5+ A1 vs. A2: P = 0.43, A1 vs. A3: P = 0.36, A2 vs. A3: P = 0.93, TH+ A1 vs. A2: P = 0.09, A1 vs. A3: P = 0.05, A2 vs. A3: P = 0.81). Furthermore, comparison between patients with high (≥ 50 U/l) and low (< 50 U/l) serum ALT revealed no significant difference in terms of either whole or sympathetic nerve fibers (Fig 4B, PGP9.5+: P = 0.88, TH+: P = 0.90). These data indicated that there was no significant relationship between the degree of inflammation and NFAR.

### Relationship between metabolic factors and NFAR

**Fig 5:**
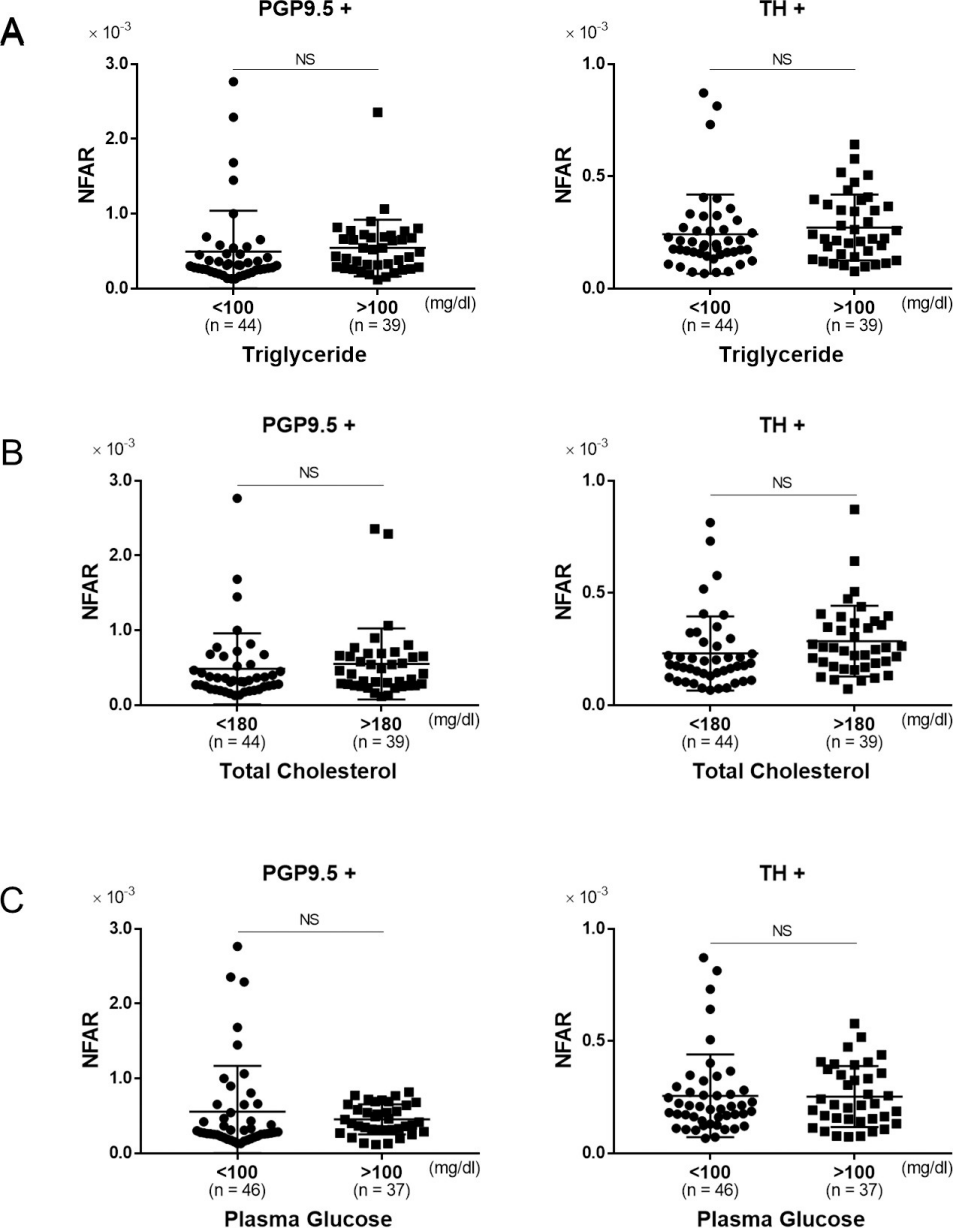
The relationship between metabolic parameters and NFAR. Comparison of NFAR for whole nerve fibers (PGP9.5+) and sympathetic nerve fibers (TH+). A: lower triglyceride level (< 100 mg/dl) vs. higher triglyceride level (≥ 100 mg/dl), B: lower total cholesterol level (< 180 mg/dl) vs. higher total cholesterol level (≥ 180 mg/dl), C: lower plasma glucose level (< 100 mg/dl) vs. higher plasma glucose level (≥ 100 mg/dl). Data were analyzed by unpaired *t* test. NS: not significant.

The association between metabolic factors and NFAR was evaluated using serum triglyceride, total cholesterol, and fasting plasma glucose as clinical parameters. There was no significant difference in NFAR between patients with a fasting serum triglyceride level above and below 100 mg/dl in terms of both of whole and sympathetic nerve fibers (Fig 5A, PGP9.5+: P = 0.99, TH+: P = 0.67). Although there was no significant difference in NFAR between these two cholesterol level groups, it tended to be higher for sympathetic nerve fibers in patients with a high cholesterol level (Fig 5B, PGP9.5+: P = 0.72, TH+: P = 0.05). There was no significant difference in NFAR between patients with high (≥ 100 mg/dl) and low (< 100 mg/dl) fasting plasma glucose level in terms of both of whole and sympathetic nerve fibers (Fig 5C, PGP9.5+: P = 0.61, TH+: P = 0.87). Thus, there appeared to be no correlation between metabolic abnormalities and intrahepatic NFAR. Therefore, the tendency for a NFAR decrease in the chronic hepatitis group suggested a possible link to fibrosis rather than inflammation or metabolic disorders.

### Chronological changes in NFAR after antiviral treatment

Liver biopsy specimens were compared before and after antiviral treatment for chronic hepatitis C in three patients: two who received interferon monotherapy and one who received peginterferon and ribavirin. In all cases, the degree of fibrosis before treatment was F3, and after treatment it became F1 to F2, indicating some degree of histological improvement.

**Fig 6:**
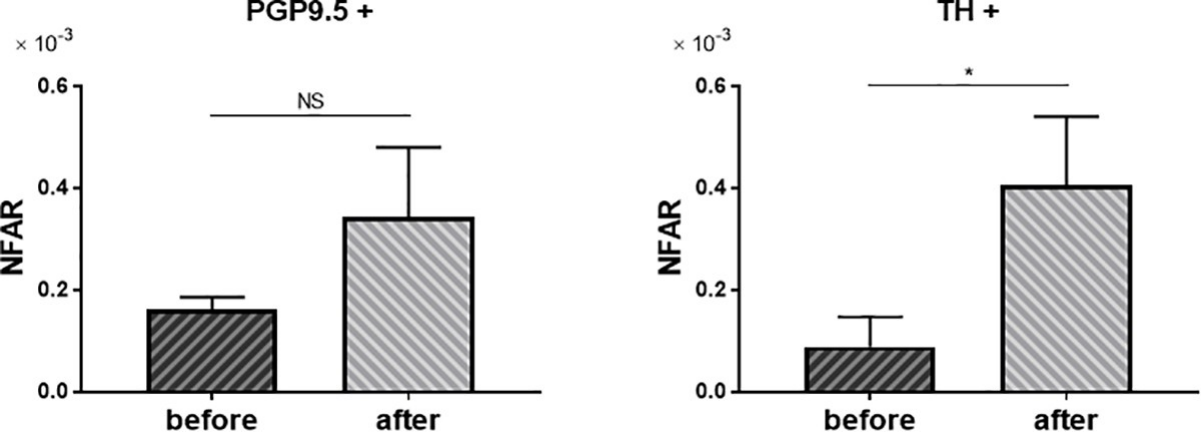
Change of NFAR after antiviral treatment. Comparison of NFAR for whole nerve fibers (PGP9.5+) and sympathetic nerve fiber (TH+). Before antiviral treatment vs. after treatment. Data were analyzed by unpaired *t* test. NS: not significant. * P = 0.02.

Comparison of NFAR before and after antiviral treatment showed that whole nerve fibers tended to increase, whereas sympathetic nerve fibers increased significantly after treatment (Fig 6, PGP+: P = 0.09, TH+: P = 0.02).

## Discussion

In the present study, we were able to demonstrate that in terms of areal ratio, intrahepatic nerve fibers were decreased in patients with chronic liver diseases. In a previous study, Nam et al. measured the number of nerve fibers in the portal area in human liver tissues, and noted a significantly decrease in cirrhosis relative to either chronic hepatitis or normal liver [31]. However, in their study, bundles of nerve fibers in the large portal tract may have been counted similarly to those in the peripheral thin fibers. In the present study, therefore, we developed a novel quantitative method for evaluation of immunostained nerve fibers in the portal area to standardize their anatomical thickness, making it possible to quantify the area of positively stained nerves as NFAR. This approach revealed that intrahepatic nerve fibers in chronic liver diseases were significantly decreased relative to those from normal liver. Among the chronic liver diseases investigated, the NFAR was low in viral hepatitis relative to that in NASH, suggesting that the role of the autonomic nervous system in the pathogenesis of chronic hepatic inflammation differs according to etiology.

**Fig 7:**
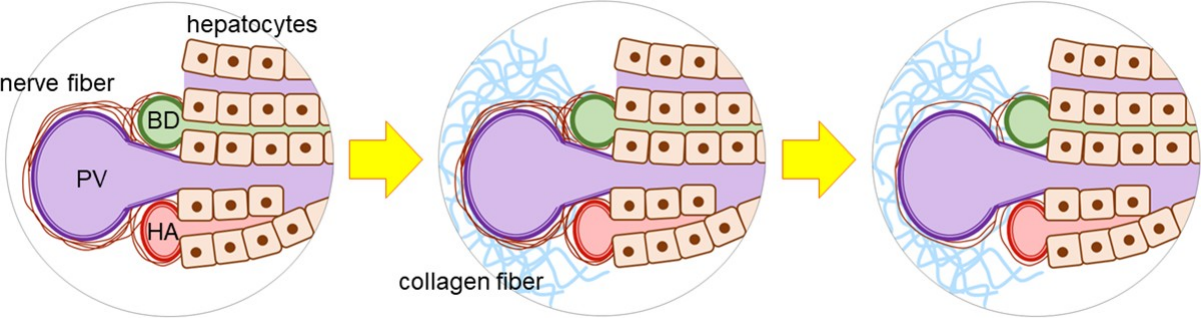
Decrease of intrahepatic nerve fibers associated with fibrosis. During the hepatic fibrosis progression, intrahepatic nerve fibers are also damaged by physical mechanism, resulting in regression of fibers. PV: portal vein, BD: bile duct, HA: hepatic artery.

In response to liver injury, hepatic stellate cells (HSCs) are activated and undergo transformation to myofibroblasts, producing collagen fibers. HSCs are believed to be under sympathetic regulation, and HSCs themselves are capable of synthesizing and releasing catecholamines such as noradrenalin [18]. Thus, it is suggested that nerve fibers have play a significant role in hepatic fibrosis. In the present study, NFARs were compared between mild fibrosis and advanced fibrosis, and were found to decrease along with the degree of hepatic fibrosis. This seems to contradict the increase of collagen fiber release in response to sympathetic stimulation. Therefore, we hypothesized a role of nerve fibers in the development of hepatic fibrosis. As fibrosis progresses as a result of chronic hepatitis, nerve fibers are also affected by physical damage, resulting in regression of fibers (Fig 7). To clarify this possibility, further research including analysis of neurotransmitters is warranted.

Although NFAR was significantly reduced in livers with advanced fibrosis relative to those with mild fibrosis, there was no significant relationship between the degree of liver inflammation and NFAR in terms of either pathological classification or the serum ALT level. This is the first study to have evaluated the distribution of nerve fibers in relation to inflammation and fibrosis. Furthermore, we have shown that fibrosis, and not inflammation, is related to nerve fiber reduction.

Antiviral treatments for viral hepatitis, such as interferon, nucleos(t)ide analogs, and DAA, have been introduced worldwide, resulting in sufficient diseases control in the majority of cases. Therefore, it is not rare to encounter cases where hepatic fibrosis has improved. However, it is still unknown whether intrahepatic nerve fibers also recover. It has been reported previously that regeneration of nerves occurs in the liver after transplantation [32–35]. Although it has been reported that liver innervation becomes reconstituted after surgical transection, it is necessary to evaluate changes in intrahepatic nerve distribution along with improvement of hepatic fibrosis. In order to investigate chronological changes in hepatic fibrosis and intrahepatic nerve fibers in identical cases, we obtained samples from three patients who had undergone liver biopsy before and after treatment for HCV. All three cases demonstrated resolution of hepatic fibrosis after viral eradication, and nerve fibers–especially sympathetic fibers–had increased. Therefore, it appears that the decrease of intrahepatic nerve fibers in diseased liver is reversible, suggesting that neurogenic adjustment for homeostasis could also be reversible.

Hepatic nerve fibers also play a role in the metabolism of carbohydrates and lipids. In this study, however, no relationship between intrahepatic nerve fibers and metabolic parameters such as the levels of fasting plasma glucose and serum triglyceride was evident. The lower serum total cholesterol levels in patients with decreased sympathetic nerve fibers might be due to impaired ability to synthesize cholesterol as hepatic fibrosis advances. To clarify the relationship between hepatic metabolism and nerve fiber distribution, it will be necessary to consider a large-scale study that takes patient background into account.

In this study, we investigated both intrahepatic sympathetic nerve fibers and whole nerve fibers including others. For evaluation of the parasympathetic fibers, immunohistochemical staining was technically difficult due to reactivity of available antibodies. It has been reported that liver regeneration after hepatectomy in rats is suppressed by vagus nerve disruption, suggesting a significant role of parasympathetic nerves in liver regeneration [36, 37]. In order to help clarify the specific physiological role of parasympathetic nerves in liver homeostasis, it will be necessary to develop novel techniques such as optogenetics or novel *in vivo* imaging.

In the present study, we examined only the analysis of immunohistochemical staining. Since the liver biopsy specimens mainly contain peripheral portal areas, the nerve fibers present in the specimens are thin and small amount. Therefore, it was considered difficult to quantify protein and gene expression of intrahepatic nerve fibers by Western blotting, ELISA, or PCR analysis. Furthermore, since all human liver biopsy specimens collected in the past at this facility were formalin-fixed, mass spectrometry could not be performed. Dynamic changes in intrahepatic nerve fibers associated with fibrosis progression or virus elimination should also be evaluated by analysis of related pathways such as MMP / TIMP, TGF-beta signaling, and stellate cell activation. However, these analyzes were also difficult as they could not be measured with formalin-fixed specimens. In the future, we are considering research using animal models. Animal models will also be able to quantify thicker nerve fiber bundles present in the central portal area and evaluate them by pathway analysis.

In the present study, it was not possible to determine whether the proportion of nerve fibers was decreasing because of expansion of the portal area in cirrhosis or whether the quantity of nerve fibers was decreasing. However, it was clear that in severe fibrosis, the nerve distribution in the portal area was relatively sparser than that in mild fibrosis. As liver disease progresses, a small amount of nerve fibers has to regulate a wider area. We believe that may contribute to the failure of liver regeneration.

Our findings suggest that intrahepatic nerve fibers are decreased in chronic hepatitis, and that the degree of impairment could be related to disease etiology. Nerve fibers were also shown to be significantly reduced along with the progression of fibrosis. Furthermore, the amounts of nerve fibers were restored after improvement of fibrosis. It also appears that fibrosis rather than inflammation activity is associated with the amount of nerve fibers in diseased liver.

## Supporting information

S1 Table Mean of positive nerve fiber area and portal area of each sample. (DOCX)
