# Supplementary material for: Intrahepatic distribution of nerve fibers and alterations due to fibrosis in diseased liver
Source: PLoS One. 2021 Apr 14;16(4):e0249556. doi: 10.1371/journal.pone.0249556 (PMC8046205; doi:10.1371/journal.pone.0249556)
Supplement: S1 Table — (DOCX) [file pone.0249556.s001.docx]

**S1 Table.** Mean of positive nerve fiber area and portal area of each sample.

| Diagnoses | PGP9.5+ | | | TH+ | | |
| --- | --- | --- | --- | --- | --- | --- |
|  | area of nerve fibers (μm2) | portal area – vessel luminal area (μm2) | NFAR (x1000) | area of nerve fibers (μm2) | portal area – vessel luminal area (μm2) | NFAR (x1000) |
| Normal | 36.7 | 19909 | 1.449 | 8.7 | 24081 | 0.323 |
| Normal | 50.3 | 21324 | 1.684 | 26 | 46466 | 0.402 |
| Normal | 25.3 | 9241 | 2.292 | 13.7 | 16565 | 0.731 |
| Normal | 15.7 | 6763 | 2.766 | 7.6 | 9313 | 0.872 |
| Normal | 70 | 35950 | 2.357 | 14 | 35700 | 0.366 |
| HBV | 7.8 | 7796 | 1 | 10.2 | 12817 | 0.81 |
| HBV | 2.7 | 18001 | 0.16 | 2.7 | 27556 | 0.11 |
| HBV | 13.8 | 39939 | 0.31 | 6.7 | 38083 | 0.19 |
| HBV | 9.3 | 41115 | 0.28 | 4.6 | 26558 | 0.16 |
| HBV | 12.6 | 36427 | 0.29 | 8 | 33539 | 0.26 |
| HBV | 12.8 | 28537 | 0.47 | 7.4 | 28384 | 0.25 |
| HBV | 62.7 | 225732 | 0.26 | 20.3 | 168501 | 0.13 |
| HBV | 27 | 50905 | 0.54 | 12 | 54818 | 0.21 |
| HBV | 19 | 87539 | 0.2 | 7.4 | 72994 | 0.1 |
| HBV | 10.8 | 64380 | 0.18 | 12.3 | 76972 | 0.16 |
| HBV | 24 | 184477 | 0.13 | 29 | 272362 | 0.11 |
| HBV | 11.8 | 43010 | 0.25 | 8.6 | 55432 | 0.17 |
| HBV | 6.7 | 38619 | 0.16 | 4.3 | 38779 | 0.11 |
| HBV | 26.9 | 60276 | 0.42 | 9.1 | 61021 | 0.16 |
| HBV | 19.9 | 142819 | 0.23 | 15 | 136803 | 0.12 |
| HBV | 6.8 | 30524 | 0.22 | 4.6 | 36265 | 0.15 |
| HBV | 22.2 | 86026 | 0.25 | 16.2 | 72314 | 0.18 |
| HBV | 9.1 | 14130 | 0.65 | 5.2 | 15001 | 0.34 |
| HBV | 9.1 | 38488 | 0.26 | 9.5 | 40135 | 0.2 |
| HBV | 78.7 | 372923 | 0.21 | 68 | 373957 | 0.17 |
| HCV | 5.2 | 17694 | 0.3 | 5.2 | 21332 | 0.26 |
| HCV | 5.8 | 39196 | 0.23 | 6.6 | 29111 | 0.26 |
| HCV | 3.5 | 5473 | 0.65 | 1.7 | 7065 | 0.23 |
| HCV | 13 | 42053 | 0.33 | 10 | 43548 | 0.25 |
| HCV | 7.2 | 23316 | 0.31 | 5.6 | 27199 | 0.2 |
| HCV | 26.3 | 40837 | 0.68 | 7.9 | 44619 | 0.22 |
| HCV | 18.8 | 71924 | 0.27 | 6.8 | 42536 | 0.17 |
| HCV | 9.6 | 38586 | 0.26 | 7.9 | 40582 | 0.17 |
| HCV | 25.9 | 58283 | 0.26 | 6.7 | 64003 | 0.13 |
| HCV | 11.5 | 109601 | 0.12 | 10.8 | 95253 | 0.11 |
| HCV | 36.8 | 51386 | 0.68 | 21 | 70081 | 0.35 |
| HCV | 12.2 | 26440 | 0.47 | 7.8 | 35560 | 0.21 |
| HCV | 46.3 | 194474 | 0.19 | 34.6 | 285887 | 0.11 |
| HCV | 23.6 | 154965 | 0.13 | 11.2 | 149416 | 0.07 |
| HCV | 10.7 | 45088 | 0.27 | 6 | 65247 | 0.1 |
| HCV | 31.7 | 137625 | 0.18 | 7.6 | 129976 | 0.07 |
| HCV | 58.9 | 208790 | 0.25 | 47.5 | 243495 | 0.18 |
| HCV | 78.8 | 207916 | 0.32 | 47.5 | 195216 | 0.19 |
| HCV | 116 | 240220 | 0.42 | 48 | 207520 | 0.21 |
| HCV | 124 | 200985 | 0.52 | 29.1 | 143759 | 0.2 |
| HCV | 46.2 | 117623 | 0.37 | 25.1 | 119777 | 0.21 |
| HCV | 29.2 | 143563 | 0.21 | 39 | 196537 | 0.21 |
| HCV | 101 | 447848 | 0.2 | 44 | 514860 | 0.12 |
| HCV | 124 | 337471 | 0.36 | 51.7 | 360105 | 0.15 |
| HCV | 71.3 | 489790 | 0.13 | 38.9 | 506454 | 0.08 |
| NASH | 3.1 | 3530 | 0.9 | 1.2 | 3926 | 0.35 |
| NASH | 4.8 | 14453 | 0.29 | 2.8 | 11600 | 0.24 |
| NASH | 2.9 | 4597 | 0.65 | 2.3 | 5581 | 0.39 |
| NASH | 4.2 | 13105 | 0.35 | 9.5 | 22650 | 0.41 |
| NASH | 9.2 | 11330 | 0.82 | 6.5 | 11345 | 0.58 |
| NASH | 9.5 | 13071 | 0.71 | 11.5 | 20859 | 0.47 |
| NASH | 10.8 | 30820 | 0.38 | 8 | 26403 | 0.28 |
| NASH | 57 | 101980 | 0.43 | 54 | 148877 | 0.3 |
| NASH | 4.7 | 17741 | 0.28 | 5.5 | 21001 | 0.18 |
| NASH | 4.7 | 5653 | 0.81 | 1.8 | 3605 | 0.51 |
| NASH | 4.8 | 10800 | 0.49 | 3.22 | 16817 | 0.19 |
| NASH | 30.5 | 49135 | 0.58 | 16.1 | 40495 | 0.36 |
| NASH | 11.4 | 13018 | 0.69 | 5.3 | 13962 | 0.31 |
| NASH | 14.2 | 26162 | 0.66 | 3.1 | 19210 | 0.22 |
| NASH | 24.9 | 33838 | 0.69 | 13.2 | 35135 | 0.4 |
| NASH | 40 | 55425 | 0.77 | 31.4 | 55191 | 0.52 |
| NASH | 9.5 | 9014 | 1.07 | 5.5 | 7720 | 0.64 |
| NASH | 11.7 | 22510 | 0.54 | 5.1 | 19116 | 0.26 |
| NASH | 6.5 | 24325 | 0.25 | 5.2 | 23725 | 0.22 |
| NASH | 18.6 | 33490 | 0.64 | 10.7 | 23299 | 0.44 |
| NASH | 8.1 | 10789 | 0.77 | 3.2 | 9012 | 0.38 |
| NASH | 15 | 30148 | 0.46 | 6.5 | 26316 | 0.22 |
| NASH | 18 | 68894 | 0.37 | 8.6 | 77660 | 0.15 |
| NASH | 25.8 | 43610 | 0.56 | 15.7 | 47205 | 0.33 |
| NASH | 14.8 | 36399 | 0.33 | 2.5 | 34891 | 0.08 |
| NASH | 63.5 | 146791 | 0.45 | 96 | 180249 | 0.33 |
| NASH | 17.3 | 19744 | 0.72 | 10.6 | 25929 | 0.41 |
| NASH | 15.7 | 33550 | 0.54 | 7.9 | 26324 | 0.27 |
| NASH | 39.5 | 238200 | 0.14 | 9 | 156840 | 0.07 |
| NASH | 54.2 | 188855 | 0.27 | 36.8 | 221125 | 0.16 |
| NASH | 68 | 214026 | 0.31 | 25 | 193714 | 0.13 |
| NASH | 84 | 257058 | 0.32 | 46 | 389436 | 0.1 |
| NASH | 28.1 | 86044 | 0.4 | 18.2 | 133975 | 0.14 |
| NASH | 79 | 179368 | 0.38 | 27 | 242890 | 0.11 |
| NASH | 70.5 | 237865 | 0.32 | 46.8 | 205910 | 0.26 |
